# Initial ratings of different types of e-cigarettes and relationships between product appeal and nicotine delivery

**DOI:** 10.1007/s00213-017-4826-z

**Published:** 2018-01-06

**Authors:** Peter Hajek, Dunja Przulj, Anna Phillips-Waller, Rebecca Anderson, Hayden McRobbie

**Affiliations:** Health and Lifestyle Research Unit, Queen Mary University of London, 2 Stayner’s Road, London, E1 4AH UK

**Keywords:** E-cigarettes, Vaping, Product characteristics, Product appeal, Nicotine, Craving

## Abstract

**Aims:**

Little is known about features of e-cigarettes (EC) that facilitate or hinder the switch from smoking to vaping. We tested eight brands of EC to determine how nicotine delivery and other product characteristics influence user’s initial reactions.

**Methods:**

Fifteen vapers tested each product after overnight abstinence from both smoking and vaping. At each session, participant’s vaped ad lib for 5 min. Blood samples were taken at baseline and at 2, 4, 6, 8, 10 and 30 min after starting vaping. Participants rated the products on a range of characteristics. The products tested included six ‘cig-a-like’ and two refillable products, one with variable voltage. We also tested participants’ own EC.

**Results:**

All products significantly reduced urges to smoke. Refillable products delivered more nicotine and received generally superior ratings in terms of craving relief, subjective nicotine delivery, throat hit and vapour production but in overall ratings, they were joined by a cig-a-like, Blu. Participants puffed more on low nicotine delivery products. Participants’ estimates of nicotine delivery from different EC were closely linked to ‘throat hit’. Nicotine delivery was less important in the initial product ratings than draw resistance, mouthpiece comfort and effects on reducing urge to smoke.

**Conclusions:**

All EC products reduced urges to smoke. Refillable products received generally more favourable ratings than ‘cig-a-likes’ with similar nicotine content. Perception of nicotine delivery was guided by throat sensations. Lower nicotine delivery was associated with more frequent puffing. The first impressions of EC products are guided less by nicotine delivery than by sensory signals.

## Introduction

E-cigarettes (EC) have the potential to generate a substantial public health benefit if there is a switch from smoking to vaping on a population scale (Hajek et al. 2014a; PHE 2015; RCP 2016). EC technology is evolving and market forces are steering product development to features that appeal to smokers and increase the rate of adoption. This has been primarily an evolutionary process with a number of innovations not taking hold and others slowly spreading. Up to now, very little formal evaluation exists to determine which EC characteristics appeal to smokers and drive EC choice.

Nicotine delivery is likely to be among the key factors that determines whether a smoker will continue to use a device (Marynak et al. 2017), but other product features are likely to play an important role, particularly during early experimentation. These may include characteristics such as product appearance, ease of use, puff resistance, ‘throat hit’, vapour volume, mouthpiece comfort, handling characteristics and e-liquid flavour and other constituents. The cost and product marketing are likely to be important too.

Determinants of consumer choice were examined so far primarily by means of consumer surveys. Although vapers often start with self-contained ‘cig-a-like’ products that are cheaper and easier to use, those who switch to vaping completely typically progress to refillable EC, which are by far the most popular product among regular vapers (ASH 2015; Cooper et al. 2016; Dawkins et al. 2013; Giovenco et al. 2014; Yingst et al. 2015) and more strongly associated with complete cessation of smoking (Hitchman et al. 2015). The likely reason is that refillable EC with stronger batteries provide better nicotine delivery (Farsalinos et al. 2014; Hajek et al. 2017). Nicotine-free EC are rarely used (Dawkins et al. 2013). Regarding flavours, individual preferences can change and vary widely (Cooper et al. 2016; Dawkins et al. 2013; Farsalinos et al. 2013) but flavours influence EC use acutely (Litt et al. 2016). In the only laboratory study evaluating user reactions that we are aware of, EC alleviated craving proportionally to their nicotine content (0, 24 or 36 mg/ml) when participants could not touch them (held in a clamp), but the discrimination was lost when they were held in hand (Van Heel et al. 2017).

Better knowledge of what drives consumer preferences could help smokers faced with the wide range of different EC products, inform the choice of EC brands for studies of the potential of EC in smoking cessation and guide further product development.

In the first study of this type, we tested eight common EC brands, together with vapers’ own devices, to determine how nicotine intake by users combined with various product characteristics determine user’s initial reactions.

## Methods

### Design

This was a crossover study involving eight popular EC products plus participants’ own EC.

### Participants

Fifteen healthy vapers who were willing to test a series of EC products were recruited via UK on-line forums of EC users and by word of mouth. Eleven were ‘dual users’ (smoking and vaping concurrently) and four had stopped smoking altogether.

### Procedures

Participants were pre-screened over the phone and attended the laboratory after overnight abstinence from both smoking and vaping.

Dual users started with a session where they smoked a cigarette of their usual brand which they brought with them. All participants, dual users and participants who only vaped, attended the next session where their own-brand EC was tested, followed by sessions testing eight different EC brands, one at a time, in the same order. Sessions were scheduled with at least 3-day ‘wash out’ periods between them.

The sessions took place between 7:30 and 9:30 a.m., depending on the participants’ availability, and took about 60 min.

Participants received £60 at the end of each session.

At each session, an intravenous line for blood sampling was placed in the forearm and the baseline blood sample was taken, after which participants were asked to smoke/vape ad lib for 5 min. Further blood samples were taken at 2, 4, 6, 8, 10 and 30 min after starting smoking/vaping.

We reported a comparison of pharmacokinetic (PK) profiles of own brand cigarette and different EC products in the 11 dual users in a separate report (Hajek et al. 2017).This study uses the full sample of 15 participants and focuses on EC product ratings.

The project was approved by the National Research Ethics Service Committee SE Coast (14/LO/0358). All participants gave written informed consent.

### Measures

Demographic and smoking history data, including Fagerstrom Test of Cigarette Dependence based on smoking prior to switching to vaping (FTCD; (Heatherton et al. 1991)) were collected at baseline. Number of puffs taken was counted during the 5-min vaping period. Urges to smoke were rated at baseline and at 5, 10, 15 and 30 min, on a scale of 1 (‘no urge at all’) to 10 (‘extreme urge’).

At the end of each session, participants were asked to rate the product on a scale of 1 to 10 regarding the following: ‘Did it relieve your urge to smoke?’ (not at all (1)—extremely well (10)); ‘How quickly did any effect happen?’ (very slowly (1)—extremely fast (10)); ‘Did you like the taste?’ (not at all (1)—extremely (10)); ‘How much nicotine do you think it delivered?’ (too little (1)—just right (5)—too much (10)); ‘Was it pleasant to use?’ (not at all (1)—extremely (10)); ‘How hard was it to draw smoke from it?’ (too easy (1)—just right (5)—too hard (10)); ‘How comfortable was the mouthpiece?’ (not at all (1)—extremely (10)); ‘How would you rate the amount of vapour it produced?’ (too little (1)—just right (5)—too much (10)); ‘How would you rate the “hit”/“scratch” at the back of your throat it provided?’ (too little (1)—just right (5)—too much (10)); ‘How likely would you be to recommend it to friends?’ (not at all (1)—extremely (10)). Questions regarding vapour and throat hit were included at a later date, so only 9 of the 15 participants completed them for all the study products.

After the final testing session, participants ranked all nine EC products, including their own brand, in order of preference. Participants had the pictures of the products in front of them during this rating to aid recall.

Blood samples were analysed at ABS Laboratories Ltd., BioPark, Broadwater Road, Welwyn Garden City, Hertfordshire, UK. PK parameters included maximum nicotine concentration (C_max_), time to the maximum (T_max_) and area under the curve (AUC_0–> 30_), a measure of total nicotine delivery over 30 min. All measures were corrected for baseline values.

### Study products

The following six 1st generation (cig-a-like) products were tested: Gamucci (16 mg/ml nicotine, ‘original taste’ [tobacco],) Blu (18 mg/ml nicotine, flavour ‘classic tobacco’), Vype (16.8 mg/ml, ‘classic tobacco flavour’, regular), E-Lites (24 mg/ml nicotine, ‘original’ [tobacco]), Puritane (20 mg/ml, ‘original’ [tobacco]) and Vuse (4.8% nicotine, ‘original’ [tobacco]). The cig-a-like products included the four EC marketed by the tobacco industry (Blu—Imperial, Vype—BAT, Puritane—Imperial Tobacco and Vuse—RJ Reynolds) and two products produced by independent manufacturers and popular in the UK (Gamucci and E-Lites (E-Lites were later acquired by Japan Tobacco)). Although some of these products are produced in different flavours, we only used the tobacco flavoured ones to minimise the problem potentially posed by individual differences in taste preferences.

We also tested one 2nd generation refillable ‘tank’ product, a popular mid-range KangerTech EVOD produced by Kanger Technology, and one 3rd generation product (a tank product which allows variable power setting), Innokin iTaste MVP 2 produced by Innokin Technology, set to 4.8 V (range 3.3–5.0 V). Both of these refillable products were used with the same 20 mg/ml nicotine e-liquid (Vermillion River ‘classic blend’ [tobacco]).

For products marketed at different strengths, nicotine concentrations were selected to be as close as possible to 20 mg/ml. Across products tested, the e-liquids contained 16–20 mg/ml of nicotine, with two exceptions. We used E-Lites with 24 mg/ml of nicotine to assess a cig-a-like product with a higher nicotine content; and Vuse was only available with strength marked as 4.8%, translating into 48 mg/ml.

The e-liquids in Gamucci and Blu contained propylene glycol; all other products contained a combination of propylene glycol and vegetable glycerol, with no specification of proportions.

Regarding the own brand EC, five participants used 1st generation products with e-liquids containing 11–16 mg/ml nicotine, four used 2nd generation products with e-liquids containing 9–24 mg/ml nicotine and six used 3rd generation products with e-liquids containing 6–12 mg/ml nicotine. Participants used a range of flavours including mixtures, with sweet (*N* = 5), fruit (*N* = 4) and tobacco (*N* = 4) flavours the most common.

### Statistical analysis

Differences between products were analysed using *t* tests with Bonferroni correction for multiple comparisons to avoid false positives due to multiple tests. A repeated measures ANOVA was used to examine the overall effects of Time and EC brand and any interactions between the two, on urges to smoke rated over the 30 min testing period. Correlational analysis was used to test for any associations between product characteristics and nicotine delivery. Multiple regression was used to assess the link between various product characteristics and objective nicotine delivery and overall product ratings. PK parameters (AUC_0–> 30_, C_max_, T_max_) were calculated using PKSolver add-in for Excel (V2.0 (Zhang et al. 2010)). Analyses were performed with SPSS v.22.

## Results

Table 1 provides baseline characteristics of the sample.

**Table 1:** Baseline characteristics (*N* = 15)

Age, mean (SD)	36.3 (11.2)
Male	86.7%
Higher education	60%
Cigarettes smoked per day before starting EC use, mean (SD)	13.4 (7.6)
FTCD before EC use, mean (SD)	3.8 (2.5)
EC cartridges used per day, mean (SD)*	1.4 (1.4)
Ml e-liquid used per day, mean (SD)**	3.7 (1.6)
No. months using EC daily, mean (SD)	19.3 (16.7)
Days EC used in last week, mean (SD)	7 (0)
Cigarettes smoked per week currently, mean (SD)***	11.2 (14.2)

*N = 6, **N = 8, data missing for one participant, ***N = 11

### Nicotine delivery and number of puffs

The number of puffs taken correlated negatively with AUC_0–> 30_ and C_max_ (*r* = − 0.80, *p* = 0.018 and *r* = −0.60, *p* = 0.115, respectively).

Refillable products generated higher nicotine levels than cig-a-like products using similar e-liquids, despite being used with fewer puffs. KangerTech and Innokin (20 mg/ml nicotine) were used with 16.8 puffs (SD = 5.3) and provided C_max_ (SD) and AUC_0–> 30_ (SD) of 11.8 (5.7) and 229 (77) while the cig-a-like products other than Vuse (16–24 mg/ml nicotine) were used with 20.4 puffs (SD = 5.6) and generated C_max_ (SD) and AUC_0–> 30_ (SD) of 9.5 (4.0) and 163 (63.4) (*p* = 0.006 for puffs, *p* = 0.029 for C_max_ and *p* = 0.001 for AUC_0–> 30_). Own brand products which were mostly refillable, provided even higher nicotine levels while using lower nicotine concentration e-liquids (see Table 2).

**Table 2:** Nicotine delivery and number of puffs from different EC products (*N* = 15)

Product	Number of puffs (SD)	C_max_ (SD)	T_max_ (range)	AUC_0–> 30_ (SD)
Own brand (6–16 mg/ml)	18 (5)	12.6 (12)	6 (2–30)	234 (197)
Blu (18 mg/ml)	20 (6)	8.4 (6)	6 (4–30)	167 (105)
Vype (16.8 mg/ml)	22 (6)	8.2 (5)	6 (4–30)	159 (88)
Puritane (20 mg/ml)	22 (10)	6.9 (5)	6 (4–30)	137 (59)
Vuse (48 mg/ml)	19 (4)	14.0 (10)	4 (2–10)	254 (113)
Gamucci (16 mg/ml)	19 (5)	10.0 (6)	6 (2–8)	185 (75)
E-Lites (24 mg/ml)	19 (6)	8.8 (6)	6 (4–30)	189 (124)
KangerTech (20 mg/ml)	17 (6)	9.8 (6)	6 (2–30)	201 (106)
Innokin (20 mg/ml)	17 (6)	11.3 (7)	6 (4–30)	230 (107)

### Effects on urges to smoke

**Fig. 1:**
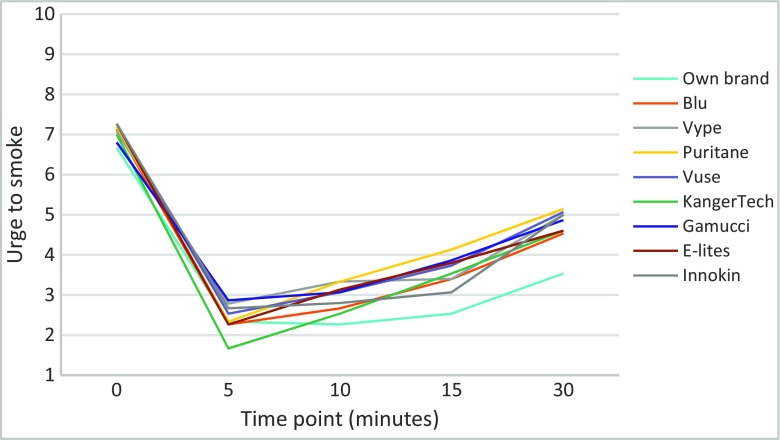
Ratings of urges to smoke when using different EC products for 5 min after overnight abstinence

Figure 1 shows the effect of different EC products on urges to smoke.

In a repeated measures ANOVA (EC brand × Time), all EC reduced urge to smoke at every time point compared to baseline (all *p*’s ≤ 0.001). There was no overall effect of EC brand nor an EC brand × Time interaction.

The tank product (KangerTech) generated a larger decrease in craving at 5 min than other products, but after adjusting for multiple comparisons, the difference was no longer significant. The own brand EC had an effect that lasted longer than the rest but when adjusted for multiple comparisons, this too became non-significant.

**Fig. 2:**
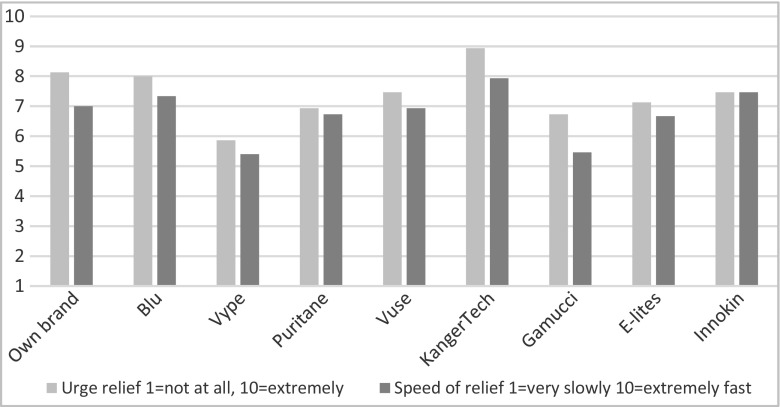
Ratings of effects of different EC brands on urge to smoke

The ratings provided at the end of each session corresponded with the momentary assessments during the vaping session, with KangerTech perceived as providing the greatest and fastest relief of urge to smoke (see Fig. 2), significantly different from Vype, Gamucci, Puritane and E-Lites (adjusted *p* = 0.03 to *p* < 0.005).

### Perception of nicotine delivery

The two advanced products (KangerTech and Innokin) were perceived as delivering the most nicotine, significantly different to Vype and Gamucci (adjusted *p*’s < 0.001) and to E-Lites (adjusted *p* = 0.028 and *p* = 0.056, respectively).

**Fig. 3:**
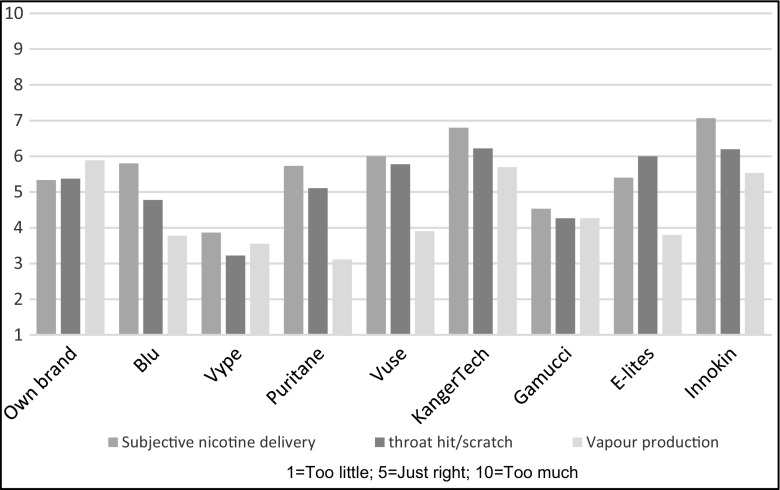
Perceptions of nicotine delivery, throat hit and vapour production

Products most often rated to be ‘just right’ in nicotine delivery were own brand (40%), followed by Vype (33%) and Gamucci (33%) (see Fig. 3).

### Throat hit

KangerTech and Innokin provided the strongest throat hit/scratch, significantly more than Vype, Gammuci and Blu (adjusted *p* < 0.02). Products most often rated to be ‘just right’ in throat hit were Puritane (44%) followed by own brand (38%) and Gamucci (33%) (see Fig. 3).

### Perception of vapour production

Own brand was rated as providing the most amount of vapour but was not significantly different to other brands when adjusted for multiple comparisons. Puritane gave the least amount of vapour and was significantly different to Innokin and KangerTech (adjusted *p* < 0.05).

Products most often rated to be ‘just right’ in vapour production were Vuse (50%), followed by own brand (44%) and Gamucci and Innokin (40%) (see Fig. 3).

### Other product characteristics: taste

**Fig. 4:**
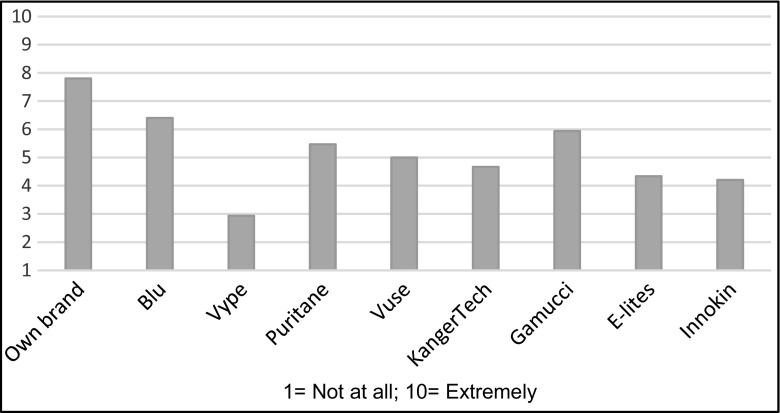
Ratings of how much participants liked the product taste

Despite the fact that all products other than own brand used tobacco flavoured e-liquids, significant differences emerged. Blu received the most favourable rating while Vype received the lowest rating, significantly below Blu, Gamucci, Puritane and E-Lites (adjusted *p* < 0.03). Own brand, which used flavours that participants selected themselves, was predictably rated the highest (see Fig. 4).

### Ease of draw

**Fig. 5:**
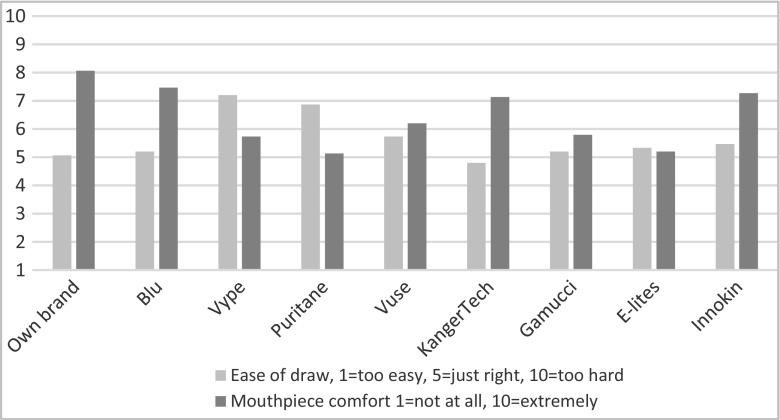
Ratings of ease of draw and mouthpiece comfort

Vype was rated as having the hardest draw, significantly different from own brand, KangerTech, Gamucci and Elites (adjusted *p* < 0.05), and marginally from Blu and Vuse (adjusted *p* = 0.05) (see Fig. 5). KangerTech had the easiest draw, significantly different from Vype and Puritane (adjusted *p* = 0.02).

Products most often rated to be ‘just right’ in ease of draw were own brand (73%), followed by Vuse and KangerTech (53%).

### Mouthpiece comfort

Own brand EC was rated as having the most comfortable mouthpiece, significantly different from E-Lites, Puritane and Gamucci (all adjusted *p*’s < 0.05) (see Fig. 5).

Of the remaining brands, Blu was rated as having the most comfortable mouthpiece, significantly better than Gammuci and E-Lites (adjusted *p* < 0.05).

### Overall ratings: pleasantness, likelihood of recommending the product to friends and retrospective ranking for overall liking

**Fig. 6:**
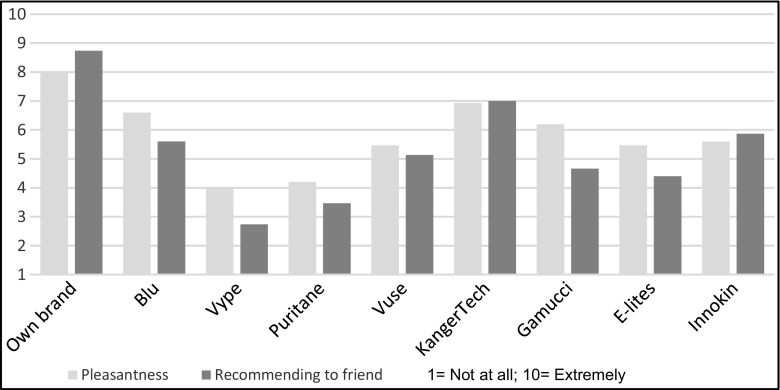
Rating of pleasantness and likelihood of recommending EC brand to friends

Own brand was rated the highest on all ratings. Among the eight other brands, KangerTech was rated the most pleasant, significantly different from Vype (adjusted *p* = 0.039) (see Fig. 6). KangerTech was also most likely to be recommended to friends (significantly better rating than Vype and Puritane, adjusted *p* < 0.001 and *p* < 0.05, respectively). Blu and Innokin also received a better rating than Vype (adjusted *p* < 0.05 and *p* = 0.05, respectively) and Blu received a better rating than Puritane (adjusted *p* < 0.05) (see Fig. 6).

**Fig. 7:**
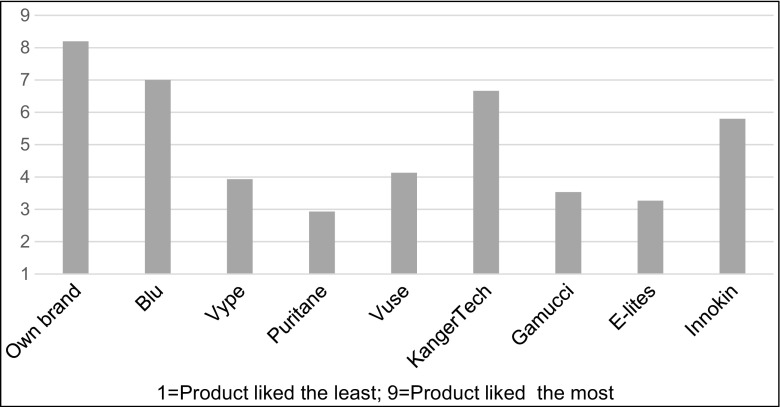
Overall rankings of EC brands

In retrospective overall liking, after own brand, Blu and KangerTech were the most liked brands, rated significantly higher than Puritane, Vype, E-Lites, Gamucci and Vuse (adjusted *p* < 0.03). Innokin was next (Fig. 7).

As all products were tested in the same order, we checked for an order effect for all three of the overall ratings. None was significant (*p* = 0.59, *p* = 0.54 and *p* = 0.31 for pleasantness, recommending products to friends, and the final ranking, respectively).

### Relationships between different product features

Of the three overall product ratings, we considered ‘recommending product to friends’ as the most relevant. The ‘pleasantness’ ratings concerned only one aspect of the product effects and the retrospective ratings relied on recalling experiences several months old.

As this was to evaluate the importance of different product characteristics on the first impressions, own brand was not included.

Table 3 shows correlations of different product features with nicotine delivery (AUC_0–> 30_) and with the ‘recommending product to friends’.

**Table 3:** Correlations between product characteristics and objective nicotine delivery and the likelihood of recommending products to friends

Product characteristic	Nicotine delivery (AUC_0–> 30_)	Recommending products to friends
Urge relief	0.35	0.80 (*p* = 0.02)
Perception of nicotine delivery	0.67	0.69
Throat hit	0.72 (*p* = 0.04)	0.54
Vapour production	0.67	0.82 (*p* = 0.01)
Taste	− 0.11	0.31
Ease of draw (‘just right’)	0.65	0.77 (*p* = 0.03)
Mouthpiece comfort	0.44	0.84 (*p* < 0.01)
Nicotine delivery (AUC_0–> 30_)	–	0.65

The correlation is not significant unless *p* value is provided

Objective nicotine delivery correlated significantly only with the rating of throat hit. (This was despite the fact that as mentioned in the ‘Methods’ section, questions regarding vapour and throat hit were included at a later date and so only 9 of the 15 participants completed them for all the study products). Recommending products to friends correlated with mouthpiece comfort, urge relief, vapour production and considering the degree of draw resistance to be ‘just right’.

Regarding other relationships between the individual variables:

Number of puffs which correlated negatively with nicotine delivery, also correlated negatively with vapour production (*r* = − 0.90, *p* = 0.003) and with throat hit (*r* = − 0.70, *p* = 0.054).

Like the objective nicotine delivery, perceived nicotine delivery also correlated significantly only with throat hit (*r* = 0.89, *p* = 0.003).

We entered the following product characteristics: ease of draw, mouthpiece comfort, vapour volume, taste, throat hit/scratch and perception of nicotine delivery into three regression models (backward stepwise regression) to determine how they relate to overall ratings of product pleasantness, the likelihood of recommending the product to a friend and the retrospective product ranking. The results need to be interpreted with caution because of the small sample size (*N* = 8).

For the retrospective ranking, significant univariate correlates included urge relief and mouthpiece comfort but the multivariate model retained mouthpiece comfort only (*b* = 0.949, *p* < 0.001). For ratings of overall pleasantness, mouthpiece comfort, taste, throat hit/scratch and perception of nicotine delivery were retained in the final model, (*b* = 1.09, *p* = 0.015; *b* = 0.48, *p* = 0.044; *b* = 1.62, *p* = 0.028 and *b* = − 1.55, *p* = 0.043, respectively.). For recommending products to a friend, the final model retained mouthpiece comfort (*b* = 0.65, *p* = 0.002), throat hit/scratch (*b* = 0.26, *p* = 0.057); and ease of draw being just right (*b* = 0.37, *p* = 0.023).

## Discussion

The study generated a range of novel findings. Before we discuss their implications, it needs to be acknowledged that the study had several limitations.

The sample was relatively small, but of the usual size for these types of studies that require an intensive participant involvement over a protracted period of time. The main drawback of a limited sample size is that the study could be expected to detect only strong trends. Negative findings need to be interpreted with caution, but a number of findings reached statistical significance. The participants were not a homogenous group in terms of their smoking (both pre- and post-dual use) and their vaping behaviour (e.g. in terms of which own brand they used and how much they used it). This means that we could only detect general effects that are applicable across a range of smokers with different needs. Most participants were male and the findings may be less generalizable to female vapers. We did not check the accuracy of the labelling of nicotine content provided by the manufacturers, but manufacturer labelling in western markets tends to be accurate (Beauval et al. 2017; Etter and Bugey 2017; Goniewicz et al. 2014). There is some indication that the proportion of PG and VG in e-liquids can affect consumer reactions (Etter 2016) but the products we tested did not provide sufficient information to evaluate this. The finding that own EC product was rated as superior to others could be influenced by the fact that all the experimental products had tobacco flavour while most participants used other flavours in their own product. All participants reported abstinence on the days of testing, but checking baseline blood results showed that one participant had somewhat elevated nicotine levels on two occasions (14.4 ng/ml and 17 mg/ml). This is likely to be due to having vaped late at night (study sessions took place between 7:30 and 9:30 am) but it would have been better to require 12-h rather than overnight abstinence. It is also important to note that different EC products were used on only one occasion. The results could be different if the products were used over a prolonged period of time. Our purpose however was to determine which product features influence the first impressions.

### Puffing frequency and nicotine intake

Puffing frequency correlated negatively with nicotine intake. This suggests that participants were making an effort to obtain more nicotine from low-delivery products. The opposite process, i.e. reducing puffing to avoid aversive effects from high delivery products, is also possible, but seems less likely. More puffs generate more vapour and more throat sensation and both of these variables correlated positively rather than negatively with recommending products to friends and so were more likely to be sought rather than avoided. As reported previously (Hajek et al. 2017), in a subsample of participants who were dual users, more puffs were taken from EC products than from cigarettes; and cigarettes delivered more nicotine than EC products (values for own cigarette were 14 puffs (SD = 4.5); C_max_ (SD) 17.9 (16); AUC_0–> 30_ (SD) 315 (155)).

If we assume that vapers increased their puffing rate to try to compensate for low nicotine delivery, it is important to note that this was not successful. Individual vapers were not achieving similar nicotine delivery from different products despite varying their puffing rate. This corresponds with a previous finding showing that novice vapers can learn to increase their nicotine intake from vaping, but the improvements are only modest (Hajek et al. 2014b). Conventional cigarettes seem to allow a much more flexible titration of nicotine delivery via, e.g. changes in the rate and depth of inhalations (Jarvis et al. 2001). Our finding suggests that existing EC products provide only a limited scope for varying nicotine intake by varying vaping topography.

### Cig-a-likes versus refillable products

Refillable EC products delivered more nicotine than cig-a-like products with similar nicotine content and received higher ratings on urge relief, perceived nicotine delivery, throat hit and vapour volume. In overall ratings however, they were joined by Blu. Blu received the highest ratings for mouthpiece comfort and for taste.

It remains unclear whether the ‘first impression’ advantages related to sensory variables remain in force after extended use. Surveys cited in the introduction suggest that over recent years, EC novices usually started on cig-a-like products but that long-term vapers use almost exclusively refillable products. It is possible that conditioned and sensory stimuli override nicotine feedback early on, but with prolonged use, vapers gravitate to products with higher nicotine delivery. Other explanations are possible, however. Vapers may find refillable products preferable and move over to them because they allow a much wider choice of flavours which for many vapers is an important consideration (Farsalinos et al. 2013); because they generate higher vapour volume which was related positively to overall product rating; and/or because for frequent users, despite the initial cost of purchasing a more advanced EC, tank products are cheaper to use than cig-a-likes.

### Effect of vaping on craving reduction, nicotine feedback and ‘throat hit’

All ECs significantly reduced urge to smoke (craving) after overnight abstinence. Previous research showed that even nicotine-free EC can have this effect (Dawkins et al. 2012; Przulj et al. 2016). The effects of conditioned sensory-motor cues and possibly also effects of distraction can be initially as important as effects of nicotine, but over time smokers seem to habituate to the signals that are no longer reinforced so they stop eliciting the initial response (McRobbie et al. 2016). It is possible that a similar mechanism applies to vapers.

When considering the substantial craving reduction that occurred largely independent of nicotine intake, it is important to note that all EC delivered some nicotine (C_max_ 7–14 ng/ml) and did this at a similar speed. Within the observed nicotine delivery range, central nicotine effects may be similar, or provide only limited experiential signals and smokers are instead clued by various conditioned sensory stimuli. Data we collected can throw some light on which sensory signals are relevant.

The only variable that correlated significantly with perceived nicotine delivery was the ‘throat hit’. Throat sensations are known to be important to vapers (Etter 2016) and they also play an important role in smoking (Rose 2006). A linear decrease in craving for cigarettes has been reported in response to anaesthesia of the mouth, pharynx and tracheobronchial airways (Rose et al. 1984). Our results suggest that the feedback from the respiratory tract is proportional to the actual nicotine intake. The throat hit correlated significantly not only with the perception of how much nicotine is being inhaled, but also with the actual nicotine intake.

### Mechanical product characteristics

Two mechanical features of EC products that we considered marginal turned out to be important. Draw resistance, which depends on the aperture of the mouthpiece, and the feel of the mouthpiece affected strongly overall product ratings. EC manufacturers should note that these two mechanical features that are easy to adjust may play a major role in the initial product appraisal.

### Conclusions

A range of EC products provide a significant reduction of urges to smoke acutely. Refillable products deliver higher nicotine levels and generate better consumer ratings in general than cig-a-likes with similar nicotine content. Lower nicotine delivery is associated with more frequent puffing. Sensations in the throat (‘throat hit’) reflect nicotine delivery and guide vapers’ perception of it. The first impressions of EC products are guided largely by sensory signals, of which draw resistance is particularly noteworthy.
